# Increasing N2200 Charge Transport Mobility to Improve Performance of All Polymer Solar Cells by Forming a Percolation Network Structure

**DOI:** 10.3389/fchem.2020.00394

**Published:** 2020-05-20

**Authors:** Ye Yan, Yadi Liu, Qiang Zhang, Yanchun Han

**Affiliations:** ^1^State Key Laboratory of Polymer Physics and Chemistry, Changchun Institute of Applied Chemistry, Chinese Academy of Sciences, Changchun, China; ^2^School of Applied Chemistry and Engineering, University of Science and Technology of China, Hefei, China

**Keywords:** molecular weight, charge transport, percolation network, solution aggregation, all-polymer solar cells, chain conformation

## Abstract

The poor electron transport ability of the polymer acceptor is one of the factors restricting the performance of all-polymer solar cells. The percolation network of conjugated polymers can promote its charge transfer. Hence, we aim to find out the critical molecular weight (MW) of N2200 on the forming of the percolation network and to improve its charge mobility and thus photovoltaic performance of J51:N2200 blend. Detailed measurements demonstrate that when the MW of N2200 is larger than 96k, a percolation network structure is formed due to the chain tangled and multi-chain aggregations. Analysis of kinetic experiments reveals that it is the memory of the N2200 long chain conformation and the extent of aggregation in solution are carried into cast films for the formation of the percolation network. Thus, the electron mobility increases from 5.58 × 10^−6^ cm^2^V^−1^s^−1^ (N2200_17k_) to 9.03 × 10^−5^ cm^2^V^−1^s^−1^ when the MW of N2200 is >96k. It led to a balance between hole and electron mobility. The μ_h_/μ_e_ decrease from 16.9 to 1.53, causing a significant enhancement in the PCEs, from 5.87 to 8.28% without additives.

## Introduction

All-polymer solar cells (all-PSCs) have attracted widespread attention due to their unique advantages, such as good morphological stability and outstanding mechanical properties (Zhou et al., 2014; Kim et al., 2015; Zhang et al., 2018; Xu et al., 2019; Yang et al., 2019a,b; Zheng et al., 2019). However, their efficient uses still cannot compete with the polymer–small molecules system (Zhu et al., 2019; Liu et al., 2020). One of the main factors restricting its performance is the poor electron transport capacity of the polymeric acceptor (Holcombe et al., 2009; Mori et al., 2011; Wetzelaer et al., 2012).

The mobility of conjugated polymers mostly increases with the increase of molecular weights, and this usually promotes PCE improvement (Bartelt et al., 2014; Fan et al., 2017; Khan et al., 2019; Li et al., 2019; Yin et al., 2019; Zhang Z. et al., 2019). Jung et al. found the tendency of face-to-face stacking to increase with an increase in the the MW of N2200 in PTB7-Th:N2200 blends (Jung et al., 2016). This stacking facilitated the free charge carrier generation, and the PCE was up to 6.14%. Marks and collaborators investigated the molecular weight effect of both PTPD3T and N2200 (Zhou et al., 2016). They found that the blend with the intermediary molecular weight of PTPD3T and N2200 can get an optimal morphology. The blend morphology, having ordered crystalline to promote charge transport, and good miscibility, provides a sufficient interface for exciton separation. Kolhe et al. (2018). produced the PNDIBS with different MWs (28.4 kDa, 57.3 kDa) and blended it with the donor PBDB-T. They found the electron mobility in the high MW blends is nearly an order of magnitude higher compared to the low MW blends. The power conversion efficiency (PCE) achieved 9.4% in all-PSCs with high-molecular-weight PNDIBS. Hoefler et al. used a different MWs PTB7-Th blend with O-IDTBR to investigate the effect of MW on the device performance (Hoefler et al., 2018). They assigned the enhanced *J*_sc_ values to the improvement of hole mobility with the increasing molecular weight of PTB7-Th. The hole mobility increased from 6.2 × 10^−3^ cm^2^ V^−1^ s^−1^ (50 kDa) to 1.05 × 10^−2^ cm^2^ V^−1^ s^−1^ (200 kDa), and the PCE increased from 8.44 to 9.57%. This kind of behavior is also described in some studies to generally originate from a higher effective conjugation length and extended interconnectivity between ordered polymer aggregates (Kline and McGehee, 2006; Gu et al., 2018; McBride et al., 2018). When the chain is long enough that it is beyond a critical MW, the interconnectivity chain can form a percolated network for supporting macroscopic charge transport. This percolation network structure can not only improve the electrical properties but also enhance the mechanical properties (Choi et al., 2019). However, this critical MW or percolation threshold has varied from study to study caused by molecular characteristics of polymers. For example, the critical MW of P3HT exists normally in the region of 20–30 kDa, and the FTAZ is below 30 kDa (Chang et al., 2006; Koch et al., 2013; Balar et al., 2019).

N2200 is one of the most studied n-type polymeric semiconductors and is broadly used as the polymer acceptor in all-polymer solar cells. Despite extensive research focused on improvements on the device performance front (Osaka et al., 2012), however, it remains a challenge to understand the mechanism of how processes can lead to efficient electronic charge transport in all-polymer solar cells (Vacha and Habuchi, 2010; Jackson et al., 2015). Within the current body of the manuscript, we have aimed at understanding how long the N2200 chain can realize the percolation network structure to optimal charge transfer pathways. The difference in the characteristic is related to the difference between the different MWs of N2200. Finally, we have correlated the evolution of such microstructures to the evolution of electronic properties in devices. The MW of N2200 used in this experiment was calculated: the number-average molecular weight of 17, 28, 57, 96, and 110 kDa, denoted as N2200_17k_, N2200_28k_, N2200_57k_, N2200_96k_, and N2200_110k_, respectively. Through a detailed study of the intrinsic feature of the N2200, we revealed the mechanism behind the N2200 percolation network structure. Thus, the relationship between the structure–property paradigms is constructed. The prepared high-performance devices have shown the importance of reasonable molecular weight selecting for performance optimization.

## Materials and Methods

### Materials

**Scheme 1:**
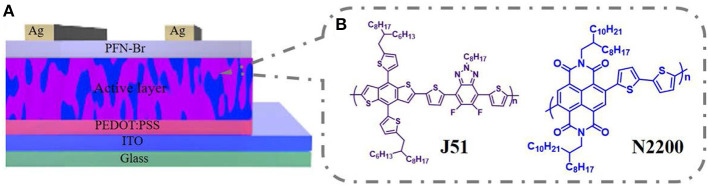
**(A)** The conventional device architecture. **(B)** The chemical structures of polymers.

J51 (M_n_ = 23 kDa, PDI = 2.1) and N2200 with different molecular weights (M_n_ = 17 kDa, PDI = 2.2; M_n_ = 28 kDa, PDI = 2.0; M_n_ = 57 kDa, PDI = 3.0; M_n_ = 96 kDa, PDI = 2.0; M_n_ = 110 kDa, PDI = 2.0) were acquired from 1-Materials company, as displayed in Scheme 1B. Solvent chloroform was purchased from Beijing Chemical Plant.

### Devices Fabrication

All-polymer solar cells were fabricated with a conventional structure of ITO/PEDOT:PSS/J51:N2200/PFN-Br/Ag, as shown in Scheme 1A. The ITO substrate was sonicated and treated in a UV ozone etching machine. The solutions were prepared by blending J51 and N2200 (2:1 w/w) in chloroform with a total concentration of 12 mg ml^−1^, stirring overnight to ensure the polymer soluble. The PBDB-T:N2200 blend system based on different MWs of N2200 is fabrication in the same condition. The active layers were spun-coated on the PEDOT:PSS-based ITO. Then, a ≈100 nm film was formed and thermally annealed at 130°C for 10 min. Lastly, a 5 nm PFN-Br and 95 nm thickness of Ag were sequentially deposited below the vacuum level of 1 × 10^−4^ Pa. The device area is 0.072 cm^2^.

### Space-Charge-Limited Current (SCLC) Measurement

The mobility is tested by the space-limited charge (SCLC) method. The structures for electron-only and hole-only were ITO/ZnO/active layer/PFN-Br/Ag and ITO/PEDOT:PSS/active layer/MoO_3_/Ag, respectively. The thickness of the active layers was ~100 nm. The film thermal annealed at 130°C for 10 min. Current–voltage (*J–V*) characteristics of the SCLC devices were tested by utilizing a Keithley 2400 SMU under dark condition in glove box filling nitrogen. The carrier mobility was extracted according to the equation of Mott–Gurney (Equation 1):

(1) $$\begin{array}{l} J=\frac{9}{8}\varepsilon_{r}\varepsilon_{0}\mu \frac{V^{2}}{d^{3}} \end{array}$$

Where *J* is the current density, ε_0_ and ε_r_ is the free space and relative permittivity, respectively. The μ is the zero-field mobility, *V* is the effective voltage (*V* = *V*_*applied*_-*V*_*bi*_-*V*_*series*_), and d is the thickness of the film.

### Characterization

The film morphology was measured by atomic force microscopy (AFM) and a transmission electron microscope (TEM). Sessile-drop measurements were done on the pure N2200 films with different MWs for water drops by using a contact angle goniometer (KRUSS GmbH Germany DO3021 Mk1). The molecular weights were tested by the gel permeation chromatography (GPC) of high-temperature in trichlorobenzene (TCB) with polystyrene as the calibration standard. The viscosities of the N2200 solutions (4 mg ml^−1^) were measured utilizing a cone-plate rheometer (BROOKFIELD DV-III ULTRA). The measurement was carried out at a constant rotor speed is 120 rpm min^−1^ at room temperature. DSC curves were tested using DSC Q2000. The heating and cooling rate is 3°C min^−1^ under nitrogen filling atmosphere. The melting enthalpy and melting temperature are read from the second heating curve, and the crystallization temperature is read from the first cycles of the cooling curve. The UV-vis absorption spectra were measured by applying a Lambda 750 absorption spectrum (Perkin-Elmer, Wellesley, MA). The instrument used for PL spectroscopy was a Jobin Yvon LabRAM HR spectrometer. The solution concentration was 0.1 mg ml^−1^. The GIXRD data were got on a Bruker D8 Discover reflector.

We tested the *J–V* characteristics curves of the devices under simulated AM 1.5G solar irradiation (100 MW cm^−2^, Keithley 2400 SMU) at a nitrogen filling glove box. The EQE results were obtained applying a QE-R 3011 instrument (Enli Tech.Co) at an ambient atmosphere.

## Results

### Photovoltaic Performance

**Figure 1:**
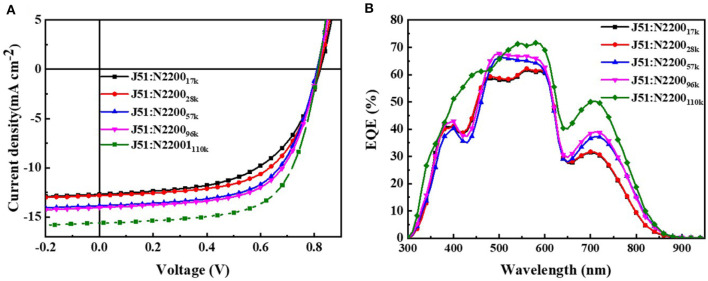
**(A)** *J-V* curves **(B)** EQE curves for J51:N2200 films based on different N2200 MWs.

To explore how the molecular weight of N2200 in blends of J51:N2200 affects the device performance, conventional-type PSCs were fabricated. The devices were processed by thermal annealing at 130°C for 10 min without solvent additives. The current density–voltage *(J–V*) curves of the devices were tested under simulated AM 1.5G, 100MW cm^−2^ illumination, as illustrated in Figure 1A. The correlative photovoltaic parameters are shown in Table 1. The J51:N2200_17k_ and J51:N2200_28k_ showed lower PCEs of 5.87 and 6.38%, respectively, with a close *J*_sc_ of about 12.8 mA cm^−2^. With the increase of the molecular weight of N2200, the *J*_sc_ and FF values increased. The *V*_oc_ value was almost unchanged (0.81–0.82 V). The *J*_sc_ and FF values of N2200_110k_ improved significantly from 12.70 mA cm^−2^ and 56.18% for J51:N2200_17k_, to 15.61 mA cm^−2^ and 64.88%. Then, a peak PCE of 8.28% was achieved, which is a 41% enhancement. External quantum efficiency (EQE) spectra of the all-PSCs are presented in Figure 1B. The *J*_sc_ got from the EQE spectra were consistent with those obtained from *J–V* measurements. The variations between them were below 6%. All the devices had a broad EQE response from 300 to 900 nm. The EQE values gradually increased with the increasing MW of N2200, particularly in the long-wavelength band of 700–900 nm assigned to N2200. The J51:N2200_110k_ blend exhibited a higher EQE response than other lower N2200 MW blend, 71%, located at 580 nm. We also fabricated the device of PBDB-T:N2200 in the same condition as shown in Figure S1 and Table S1. The same trend can found in PBDB-T:N2200 system. A peak PCE of 7.98% was achieved for PBDB-T:N2200_110k_. It is much larger than 5.74% for PBDB-T:N2200_17k_. The *J*_sc_ values improved significantly from 10.55 mA cm^−2^ for PBDB-T:N2200_17k_ to 16.17 mA cm^−2^ for PBDB-T:N2200_110k_. Hence, the *J*_sc_ is a strong N2200 molecular weight dependent, increasing with N2200 MWs.

**Table 1:** Summary of photovoltaic performance for J51:N2200 and relevant parameters of carrier mobility for blend film.

**Donor:acceptor**	***V*_**O*c***_ (V)**	***J*_**SC**_ (mA cm^**−2**^)**	**FF (%)**	**PCE (%)**	**μ_**H**_ (cm^**2**^V^**−1**^s^**−1**^)**	**μ_**E**_ (cm^**2**^V^**−1**^s^**−1**^)**	**μ_**H/**_μ_**E**_**
J51:N2200_17k_	0.82	12.70	56.18	5.87	9.43 × 10^−5^	5.58 × 10^−6^	16.9
J51:N2200_28k_	0.82	12.82	60.80	6.38	1.14 × 10^−4^	7.55 × 10^−6^	15.1
J51:N2200_57k_	0.81	13.85	62.56	7.02	4.67 × 10^−5^	1.64 × 10^−5^	2.85
J51:N2200_96k_	0.81	14.01	61.62	7.22	6.07 × 10^−5^	2.85 × 10^−5^	2.13
J51:N2200_110k_	0.82	15.61	64.88	8.28	1.38 × 10^−4^	9.03 × 10^−5^	1.53

*(Average values are obtained from 10 devices)*.

### Charge Transport Ability

**Figure 2:**
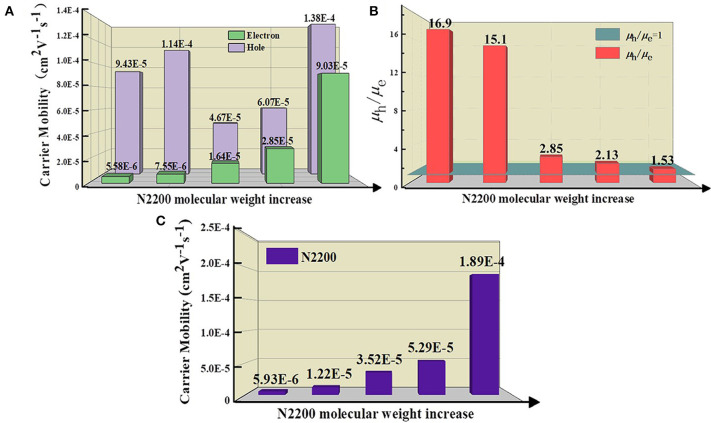
**(A)** The hole and electron mobility **(B)** The ratio value of hole and electron mobility for J51:N2200 films based on different N2200 MWs. **(C)** The electron mobility of pure N2200 with different MWs.

To further study the cause of the enhanced *J*_sc_ in the devices, we tested the ability of charge transport. The mobility is examined by the space charge-limited current (SCLC) method, as illustrated in Figure S2. The detailed parameters are listed in Table 1. The increase of MW of N2200 improved the electron mobility (μ_e_) values of the PSCs, as displayed in Figure 2A. The μ_e_ was 16 times larger in the N2200_110k_ blends. As the MW of N2200 increases, the mobility of electrons and holes is more balanced, and the ratio between hole mobility (μ_h_) and μ_e_ decreases from 16.9 to 1.53, as shown in Figure 2B. The results of the SCLC mobility indicated the enhanced PCEs of the blend devices with different MWs of N2200 are due to improving the ability of charge transport. We speculated that high MW of N2200 molecules is sufficient to form percolation pathways to facilitate electron transport. To prove our conjecture, the μ_e_ and μ_h_ of the pure components were measured (Figure 2C, Figure S3). As with the blend system, the hole mobility of J51 is 2.29 × 10^−4^ cm^2^V^−1^s^−1^, which is higher than the electron mobility of different MWs of N2200 in this work. Meanwhile, the μ_e_ increases with the N2200 MW increasing. The μ_e_ was 1.89 × 10^−4^ cm^2^V^−1^s^−1^ in N2200_110k_ devices, which is 32 times the improvement compared to N2200_17k_. The electron mobility of pure N2200 is gradually increased when increasing the MWs, getting closer to the pure J51. The mobility data of pure component provide a solid support for the high MW N2200 forming a percolation network structure to improve the ability of charge transport.

### A Percolation Network Phase Separation Structure

**Figure 3:**
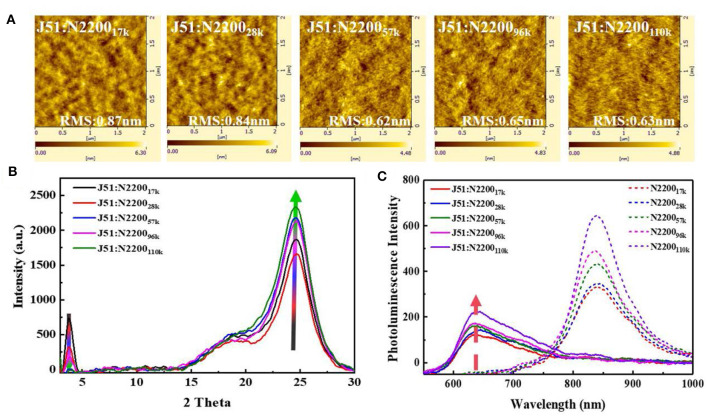
**(A)** The AFM height images **(B)** GIXRD spectra of J51:N2200 films with different N2200 MWs. **(C)** PL spectra of the film (excited at 532 nm), where the solid line representative blend films, dash line representative pure films of N2200.

We compared the morphology of the blend films as a function of MW of N2200 Figure 3A. As the MW increased, the surface roughness of the film gradually reduced from 0.87 to 0.63 nm. The phase separation size of J51:N2200_17k_ and J51:N2200_28k_ are significantly larger than the high-molecular-weight blend system. Furthermore, the fiber structure can be observed obviously in both J51:N2200_110k_ blend and the pure N2200_110k_ (Figure S4). The crystallinity of the blend system is shown in Figure 3B. With the increase of the MW of N2200, the intensity of the (100) peak of N2200 at 3.75° in the blended film gradually weakened, while the π-π stacking direction of (010) both N2200 at 22.5° and J51 at 24.5° was enhanced (Gao et al., 2016). This result indicates that the blend is more preferentially face-on orientated when the N2200 MW increases. The fluorescence spectrum tested to compare the purity of the phase domain, as revealed in Figure 3C. For pure component N2200, the intensity at 838 nm increases with increasing MW. After blending with the donor J51, the fluorescence intensity decreased sharply. The intensity of J51 at 637 nm increased with the increase of N2200 MW. This means that the phase purity of J51 in the blend system is strengthened as the MW of N2200 increases.

Taken together, these results indicate that the N2200_110k_ is long enough to form a percolation network structure compared to other MWs. This percolation network can produce an optimal blend morphology with smaller size of phase separation and purer phase domain purity and the stronger tendency of face-on orientation to realize both high *J*_sc_ and PCE.

### The Memory of the Long Chain Conformation and the Extent of Aggregation in Solution Are Carried into Cast Films for the Formation of the Percolation Network

To understand why N2200_110k_ can form a percolation network, the chain structural characteristics of different MWs were investigated by the following detailed measurements.

**Figure 4:**
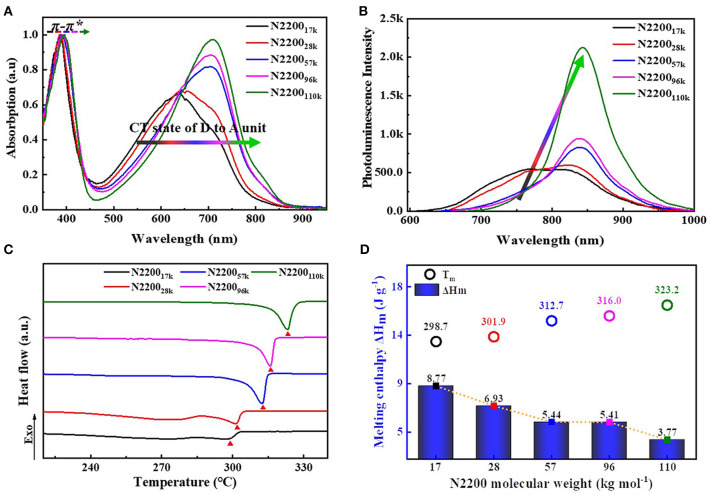
**(A)** Normalized UV-vis absorption **(B)** PL spectra in chloroform solutions (0.1 mg/ml). **(C)** The second heating cycles of DSC curves **(D)** Summary of melting temperature (*T*_m_) and melting enthalpy (Δ*H*_m_) with different N2200 MWs.

Figure 4A is the solution of the absorption spectra of different MWs of N2200. The N2200 solution concentration is 0.1 mg ml^−1^. All samples revealed two bands: high-energy bands are ascribed to π-π^*^ transition (nearly 390 nm), and low-energy bands are assigned to intramolecular charge transfer transition (500–800 nm). The absorption of the N2200_17k_ and N2200_28k_ revealed a broad and featureless band centered at about 640 nm. The appearance of the low-energy bands is indicative of the fine structure when the MWs reach 57k and 96k. In N2200_110k_, the absorption spectra redshift, with a peak at 710 nm and a shoulder at ~815 nm.

Changes of fluorescence spectra are in analogy to the absorption spectra displayed in Figure 4B. As the molecular weight increases from 17k to 110k, it leads to a pronounced redshift of the emission maximum. The emission spectra in the N2200_17k_ and N2200_28k_ are rather complex, showing distinct peaks at 760 and 820 nm. The spectrum in N2200_110k_ exhibits a more structured emission, with the peak center at 843 nm. Furthermore, the lower energy band intensity increases with the increasing MWs of N2200. The one of N2200_110k_ is 300% stronger than that of N2200_17k_.

This pronounced changes in absorption and emission are similar to Neher and co-workers' results about the spectra of N2200 in different solvents (Schubert et al., 2012; Steyrleuthner et al., 2012). They dissolved two batches N2200 (MW = 29 and 526 kDa, convert to our experiments) in toluene (Tol), trichlorobenzene (TCB), and chloronaphthalene (CN) (Steyrleuthner et al., 2012). Notably, in CN and Tol, the spectrum was almost unchanged with MW. However, significant changes occurred in TCB as the MW changed. Therefore, we concluded that the effect of molecular weight on spectral chromophores mainly occurs in a moderately good solvent, such as chloroform and TCB. It is an intrachain phenomenon and related to different conformations of individual chains.

The melting temperature (*T*_m_), melting enthalpy (Δ*H*_m_), and crystallization temperature (*T*_c_) of N2200 with different MWs were characterized by thermal analysis DSC (summarized in Table 2, Figure S5). The second heating curve of DSC was selected for the measurement of melting temperature, as shown in Figure 4C. The changes of *T*_m_ and Δ*H*_m_ as a function of MW are shown in Figure 4D. As the molecular weight increased from 17k to 110k, the *T*_m_ increased from 298.7 to 323.2°C. The Δ*H*_m_ also has a molecular weight dependence. The Δ*H*_m_ decreases from 8.77 to 3.77 J g^−1^ with an increase of MW. It is because that N2200_17k_ has a shorter chain length, forming a chain extend crystals with less entangled structure. The entanglements happen in a longer chain of N2200_110k_, hindering the crystallization process and resulting in a reduced crystallinity.

**Table 2:** Characteristics of N2200 with different MWs.

	**Mn (kDa)**	**PDI**	**λ_MAX_ (nm)**	***T*_**M**_ (°C)**	***T*_**C**_ (°C)**	**Δ*H*_**M**_ (J/g)**
N2200_17k_	17	2.2	636	298.7	281.6	8.77
N2200_28k_	28	2.0	652	301.9	285.5	6.93
N2200_57k_	57	3.0	705	312.7	295.2	5.44
N2200_96k_	96	2.0	705	316.0	298.6	5.41
N2200_110k_	110	2.0	710	323.2	300.9	3.77

**Figure 5:**
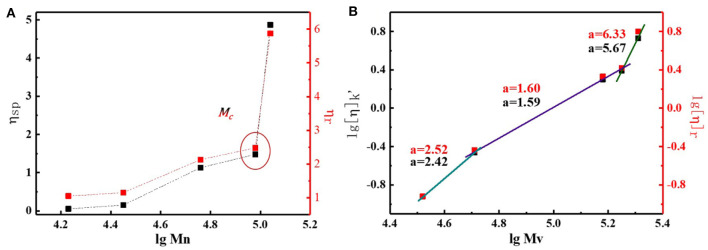
**(A)** The change of relative viscosity and specific viscosity with N2200 number-average molecular weights. **(B)** Change of intrinsic viscosity with viscosity-average molecular weights.

By studying the basic properties of different MWs N2200, we found that the chain conformation plays a key role in determining the phase separation structure. Though a direct and quantitative characterization of chain conformation remains a challenge, prediction of the backbone planarity and rigidity can provide the key steps to help our understanding of the chain conformation characteristic. Here, we hoped to utilize a power law relationship that exists between the intrinsic viscosity and the molecular weight to obtain information of chain conformation. The corresponding viscosity values are summarized in Table S2. The viscosity of the solvent chloroform was 0.61 mPa S, and the deviation from the conventional value was 8%. This discrepancy could be attributed to different test methods. Figure 5A shows the change of relative viscosity and specific viscosity with N2200 number-average molecular weight. In chloroform, the η_sp_ of the N2200 increased from 0.05 to 1.48 when the MW increase from 17k to 96k. Strikingly, the η_sp_ dramatically increased when the molecular weight reached 110k. Though the Mn of N2200_110k_ increase merely 6-fold than the N2200_17k_, the η_sp_ yielded a 97-fold increase. The η_r_ values follow the same trend. The marked increase in solution viscosity is caused by polymer chains entangling when the MW achieved the critical molecular weight (Na et al., 2019). The critical molecular weight range is consistent with the literature (Choi et al., 2019). According to the Mark–Houwink formula where [η] = KMη^a^, in a certain molecular weight range, the values of the index can reflect the chain conformation. Here, we used the relative viscosity (Equation 2) and specific viscosity (Equation 3) to calculate the intrinsic viscosity of the solution. The change of intrinsic viscosity with the viscosity-average molecular weight (M_v_) is plotted in Figure 5B. The k' in Equation 3 is the interaction parameter between N2200 and chloroform, and its value is shown in Figure S6. The slope of the curve is a, which is obtained by taking the logarithm of the molecular weight and the intrinsic viscosity.

(2) $$\begin{array}{l} \left[\eta \right]=0.25(\eta_{r}-1+3\text{ln}\eta_{\text{r}}/\text{c} \end{array}$$

(3) $$\begin{array}{l} {\text{η}}_{\text{sp}}/c=\left[\text{η}\right]+{\text{k}}^{\text{′}}{\left[\text{η}\right]}^{2}c \end{array}$$

The value obtained by the two methods are basically the same. The slope of the curve can be divided into three molecular weight intervals. At N2200_17k_ and N2200_28k_, the value of a is about 2.4. For rigid polymers, the value of a is close to 2. Therefore, the chain behaved as a rigid short rod-like structure in MWs 17k~28k. The value of a decrease to 1.6, when the molecular weight increases to 57k and 96k. The polymer chain is a rigid chain structure. It is worth noting that the value of a is about six at N2200_110k_, which is far away from the theoretical value of a. In the same polymer-solvent system, the longer polymer chain is, the greater the tendency is to bend and entangle in solution. The chain may form a cylindrical conformation (Hu et al., 2000; Adachi et al., 2010). The range of a with molecular weight is consistent with the spectrum. It firmly proves that the conformation change is the main reason for the change of spectrum. Meanwhile, the N2200_110k_ has arrived at a critical MW.

**Figure 6:**
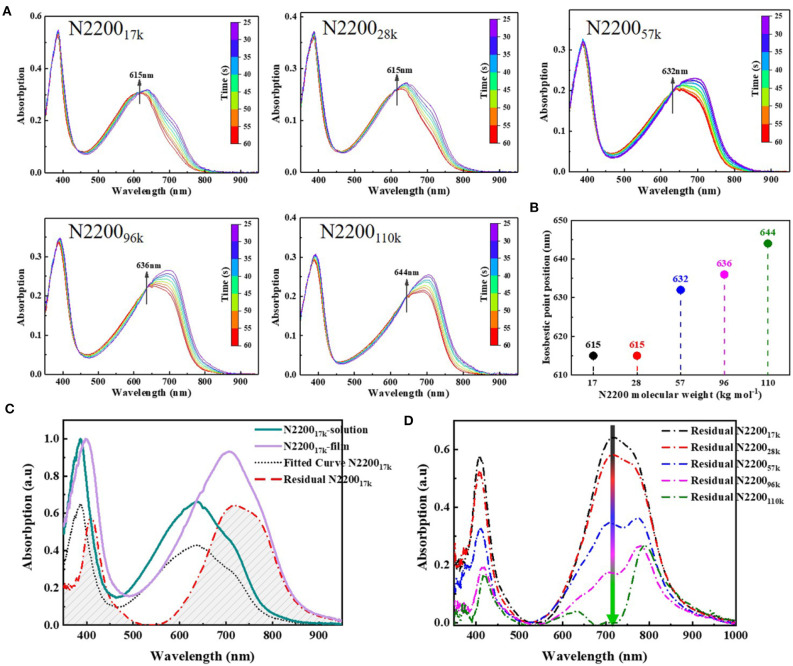
**(A)** Variable-temperature UV-Vis absorption spectra of N2200 with different MWs. **(B)** Plot of isobestic point peak position vs. N2200 MWs. **(C)** Processing of film and solution absorption spectra, the green solid line is the normalized pristine solution spectra of N2200_17k_, the purple solid line is the normalized pristine film spectra of N2200_17k_, the black dot line is solution spectra after fitting pristine solution spectra to film spectra and the red dotted line is the spectra film spectra subtracted the fitted solution spectra. **(D)** Summary of the contribution of aggregation during film formation at different N2200 MWs.

The chain conformations greatly impact the aggregation behavior, which is important for charge transport in conjugated polymers (Liu et al., 2019; Zhang Q. et al., 2019). For this reason, we next analyzed in detail the aggregate behavior caused by conformation changes in the solution and film formation. The aggregation is a temperature-controlled process that is driven by a thermodynamic order–disorder transition (Kohler et al., 2012; Panzer et al., 2014, 2017; Reichenberger et al., 2018). Here, we used temperature-dependent absorption spectroscopy to study this order–disorder transition with different N2200 MWs. The low-energy band redshifted, and intensity grew alongside the reduction in temperature, as shown in Figure 6A. All samples gave rise to an isosbestic point. The isosbestic point indicates an equilibrium between two components in the solution: one corresponds to the planar structure of the chain with a longer and ordered conjugation length and another a disordered phase. The position of the isosbestic point is plotted as a function of MWs, as illustrated in Figure 6B. The conformational degree of freedom reduced with the reduction of temperature, causing the enthalpy upon planarization to become dominant, and the entropy contributed decreased. We therefore interpret from the data shown in Figure 6B that the isosbestic point gradually redshifted with an increase in the MW, and chain backbones tended to be more planar, causing effective electronic delocalization. Meanwhile, the solution contained larger multi-chain aggregates with an increase in the MW. These aggregates were more thermodynamically advantageous, owing to the lower the energy between the transform ordered and disordered phases.

To comprehensively understand the effect of aggregation from solution to film state, we subsequently tested the absorption spectra of films of different N2200 MWs. Using the Franck–Condon analysis of the absorption (Ho et al., 2001), the contribution of the solution state in the film is subtracted to obtain the aggregation during film formation (Jiao et al., 2019). Taking N2200_17k_ as an example (Figure 6C), the data of other MWs are listed in Figure S7. The aggregation during film formation with different N2200 molecular weights is shown in Figure 6D. As the MW increases from 17k to 110k, the spectral intensity gradually decreases in the entire absorption range. The intensity of the π-π ^*^ transition decreases, and this is accompanied by the peak position being redshifted from 409 to 420 nm. Meanwhile, the CT state transition peak at 710 nm showed a dramatic decline with MW increases. These changes indicate the ordered structures have existed in solution and are preserved during the drying process. It has only a few rearrangements of polymer chains in the process of drying in high MW N2200, whereas the lower MW chains transform from solution to film happens chain aggregation within some coils or agglomerates. We interpreted from this data that the high-molecular-weight N2200 has longer active repeat units (Fauvell et al., 2016), and the longer chain length leads to a slow chain movement rate with the chain rearrangement appearing insufficient.

The detailed information obtained was used to form a complete and consistent picture of the chain conformation and aggregates characteristics vary as a function of N2200 molecular weights.

Next, to verify whether the structural characteristics of the molecular chain of N2200 still exists in the blend system, we characterized the aggregation of the blend solution and the film-forming drying process. We aimed to build a complete image of the chain structure and device performance.

**Figure 7:**
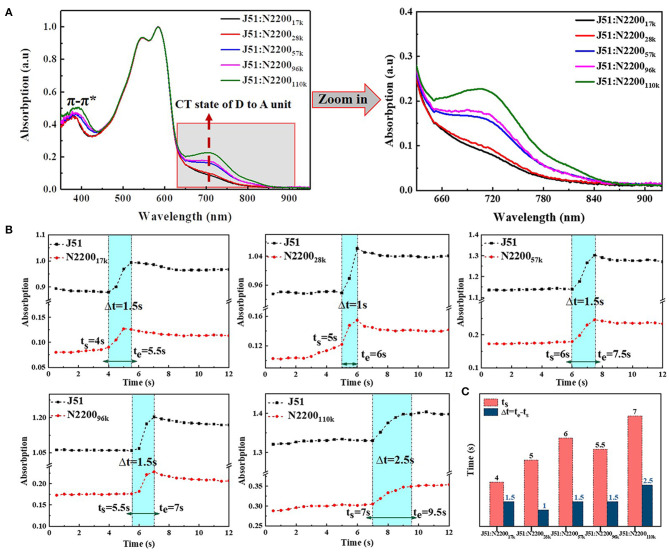
**(A)** Normalized UV-vis absorption spectra of blend in chloroform solution (concentration is 0.1 mg/ml). **(B)** The absorption intensity at 596 and 710 nm change with time at different N2200 MWs blend systems. **(C)** Summary of the start time (t_s_) and the entire time (Δt = t_e_-t_s_) of the second stage in solvent evaporation.

The normalized solution absorption spectrum of different N2200 MWs and J51 blend system, as shown in Figure 7A. The two absorption peaks of J51 for 545 nm and 585 nm in the different MWs N2200 blend systems are coincident. However, the π-π^*^ and the CT state transition peak of N2200 have strong molecular weight dependence. The π-π^*^ transition peak increased with increasing MW of N2200, and the peak position was redshifted from 380 to 391 nm. By enlarging the absorption wavelength of Figure 7A from 650 to 900 nm, we can see that the CT state transition peak intensity of N2200 at 710 nm gradually increases with the increase of the MW of N2200. It corresponds to the improved charge transfer between adjacent electron-donating NDI and the electron-withdrawing T2 unit. This reflects that the aggregates increase in solution when the molecular weight of N2200 increases. The change region of MW in the blend solution absorption intensity is in good agreement with the aforementioned chain conformation and aggregates. We thus concluded that the properties of N2200 in the pure component can hold in the blended solution.

The non-equilibrium assembly occurs during the fabrication process, which is critical to determining thin film morphology across the length scale (Patel and Diao, 2018). We therefore used *in situ* time-dependent absorption spectroscopy to study the effect of aggregation on the solution drying process. The original data are shown in Figure S8. The position of aggregation of J51 at 596 nm and the CT state transition of N2200 at 710 nm was plotted as a function of time with different N2200 MWs, shown in Figure 7B. The solvent evaporation in the film formation process is mainly divided into three periods in the spectra (Wang et al., 2010; van Franeker et al., 2015; Engmann et al., 2016; Yan et al., 2019). The first stage (dissolved state) is where the solvent molecules evaporate rapidly, and the spectral intensity remains unchanged. The second stage (nucleation and growth) is where the solvent content decreases, and the evaporate rate slows. The solution concentration increases to reach saturated solubility, and the polymer chain begins nucleation and growth. The spectral intensity gradually increases. The rate of solvent evaporates is related to the size and shape of the polymer molecule in this stage. The third stage (film formation complete) is where the solvent is completely evaporated, the film formation process is over, and the spectral intensity remains unchanged. We compared the start time (t_s_) and the entire time (Δt = t_e_-t_s_) of the second stage in solvent evaporation of blend system with different MWs N2200, as shown in Figure 7C. The t_s_ increased from 4 s at 17k to 7 s at 110k. This phenomenon is due to the difference in the viscosity of different MWs N2200. The low viscosity N2200 accelerates the chloroform solvent evaporation on the film surface, while the high-molecular-weight N2200 has a large viscosity. The slow convection rate with air makes the solvent evaporation rate slow. Furthermore, the Δt is almost the same when the MW <110k, and the Δt increased from 1.5 to 2.5 s when MW reached 110k. The Δt of a molecular weight reaching the critical MW is stronger than other MWs; the slower solution drying kinetics were explained by the slow diffusion rate of multi-chain aggregates. The data provided strong support for the formation of an effective percolation network in the N2200_110k_ blend system.

**Figure 8:**
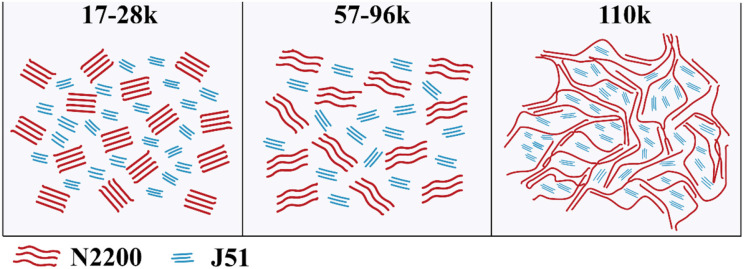
Illustration of the chain behavior effects on the resulting morphologies of J51:N2200 films with different molecular weights, low MW **(Left)**, medium MW **(Middle)**, and high MW **(Right)**.

From the above, we can entirely understand the complete picture of the relationship between the structure of the MW of N2200 and the property of J51:N2200. The chain conformation depends strongly upon the MW because of the effect MW has on the chain contour length. According to the value of the power index, which we retrieved from the viscosity test in Figure 5, we can divide the conformation into three regions. As shown in Figure 8 (left), the persistence length resulted in a highly rigid backbone, which behaved rigid and rod-like in the low molecular weight polymer (17k−28k for N2200). Though the extended chain improves the crystallinity for low MW of N2200, the short chain makes for poor connectivity between adjacent crystalizes. Furthermore, there is good solubility and the high degree freedom of chain movement in film formation; this resulted in overmixing between donor and acceptor. The blended films form a poor domain purity. The insufficient charge transport and overmuch mixed domain lead to a low power conversion efficiency. In the region of medium MW (57k−96k for N2200), the contour length of the chain increases, and this is presented as a coil-like rod as shown in Figure 8 (middle). The solution aggregation increases with the decrease in solubility. It enhances the probability of intermolecular collision among individual polymer chains. The intermolecular interaction of N2200 therefore increases. The slightly higher domain purity makes a middle the power conversion efficiency. Strikingly, the polymer of N2200_110k_ shows greater flexibility, as shown in Figure 8 (right). From the high viscosity value for N2200_110k_, it is not a surprise that 110k reaches a threshold forming a percolation network. The solution has a strong aggregation tendency in N2200_110k_. Combined with the film formation kinetics experiment, we believe that the pre-aggregation caused by the N2200 network in solution acts as a pristine crystal nucleus. The network structure of N2200 furthers collapse and self-assembly in the process of film formation. Hence, high sufficient connectivity between crystallizes enhances the electron transport. Finally, an 8.28% PCE was realized.

## Conclusion

By studying the characteristics of N2200 materials with different MWs, we found that, as the chain length increases, the chain conformation of N2200 can be divided into three ranges. The chain behaves like a rigid-rod structure for N2200_17k_ and N2200_28k_. The molecular rigidity is weakened, and a rigid chain structure is observed for N2200_57k_ and N2200_96k_. When MW is at N2200_110k_, which is larger than the critical MW, the molecular chains are tangled. Multi-chain aggregations appear when the MW is larger than the critical one, which providing opportunities for the formation of percolation networks. This structure can effectively decrease the size of phase separation and improve the purity of the phase domain and the tendency of face-on orientation. It leads to an effective charge transport pathway. Thus, the charge transport between electron and hole mobility is more balanced. The power conversion efficiency is increased from 5.87% for J51:N2200_17k_ to 8.28% for J51:N2200_110k_. This research can guide for the fabrication of high-performance all-polymer solar cells by choosing the suitable MW of polymer, which is distinguishable from a small molecule, to achieve percolation network structure.

## Author Contributions

YY conceived and performed the experiments and wrote the manuscript. YL helped to test the performances of the device. QZ discussed the experimental details. YH directed this work and revised the manuscript.

## Conflict of Interest

The authors declare that the research was conducted in the absence of any commercial or financial relationships that could be construed as a potential conflict of interest.
