# Supplementary material for: Increasing N2200 Charge Transport Mobility to Improve Performance of All Polymer Solar Cells by Forming a Percolation Network Structure
Source: Front Chem. 2020 May 20;8:394. doi: 10.3389/fchem.2020.00394 (PMC7251163; doi:10.3389/fchem.2020.00394)
Supplement: Supplementary file 1 [file Data_Sheet_1.docx]

**Supporting Information**

**Increasing N2200 Charge Transport Mobility to Improve Performance of All Polymer Solar Cells by Forming a Percolation Network Structure**

*Ye Yan^1^**^,2^*, *Yadi Liu^1,2^*, *Qiang Zhang^1,^ ** *and Yanchun Han^1,2^ **

*^1^State Key Laboratory of Polymer Physics and Chemistry, Changchun Institute of Applied Chemistry, Chinese Academy of Sciences, Changchun 130022, P. R. China;*

^2^ *University of Science and Technology of China, Hefei,*

*230026, P. R. China.*

Tel: 86-431-85262175, Fax: 86-431-85262126,

Email: [zhqawh@ciac.ac.cn](mailto:zhqawh@ciac.ac.cn)，[ychan@ciac.ac.cn](mailto:ychan@ciac.ac.cn)

* To whom correspondence should be addressed


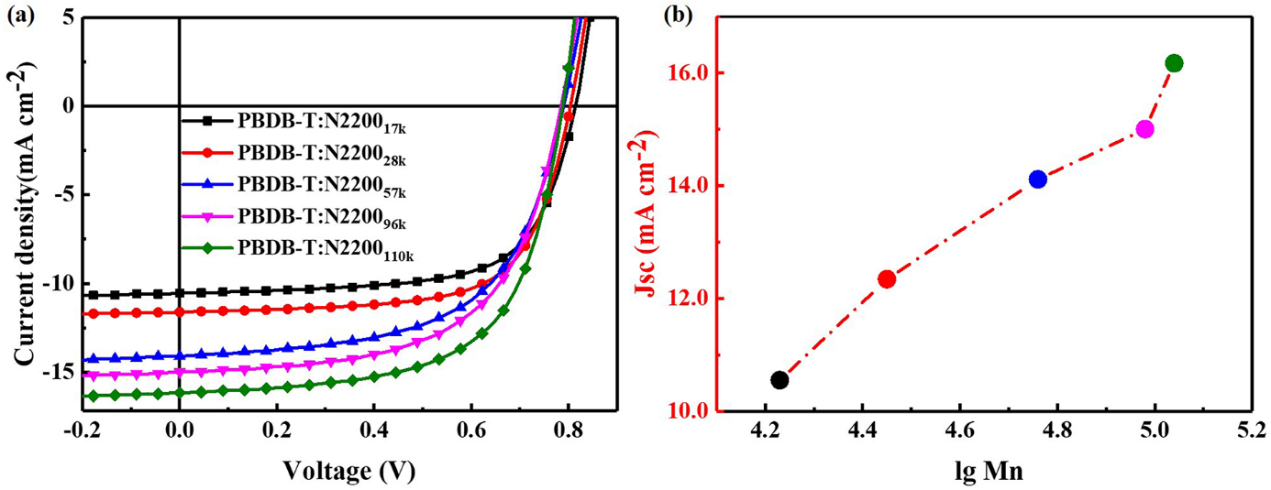


**Figure 1.** (a) *J-V* curves for PBDB-T:N2200 films based on different N2200 MWs (b) The change of short-circuit current as a function of the N2200 MWs, the red dashed line for guiding to the eye.

**Table 1.** Summary of photovoltaic performance for PBDB-T:N2200 based on different N2200 MWs. (Average values are obtained from 10 devices)

| **donor:acceptor** | ***V*_oc_**  **(V)** | ***J*_sc_**  **(mA cm^-2^)** | **FF**  **(%)** | **PCE**  **(%)** |
| --- | --- | --- | --- | --- |
| PBDB-T:N2200_17k_ | 0.81 | 10.55 | 66.80 | 5.74 |
| PBDB-T:N2200_28k_ | 0.80 | 12.34 | 64.11 | 6.37 |
| PBDB-T:N2200_57k_ | 0.79 | 14.11 | 58.95 | 6.57 |
| PBDB-T:N2200_96k_ | 0.79 | 15.00 | 59.29 | 6.99 |
| PBDB-T:N2200_110k_ | 0.79 | 16.17 | 62.39 | 7.98 |


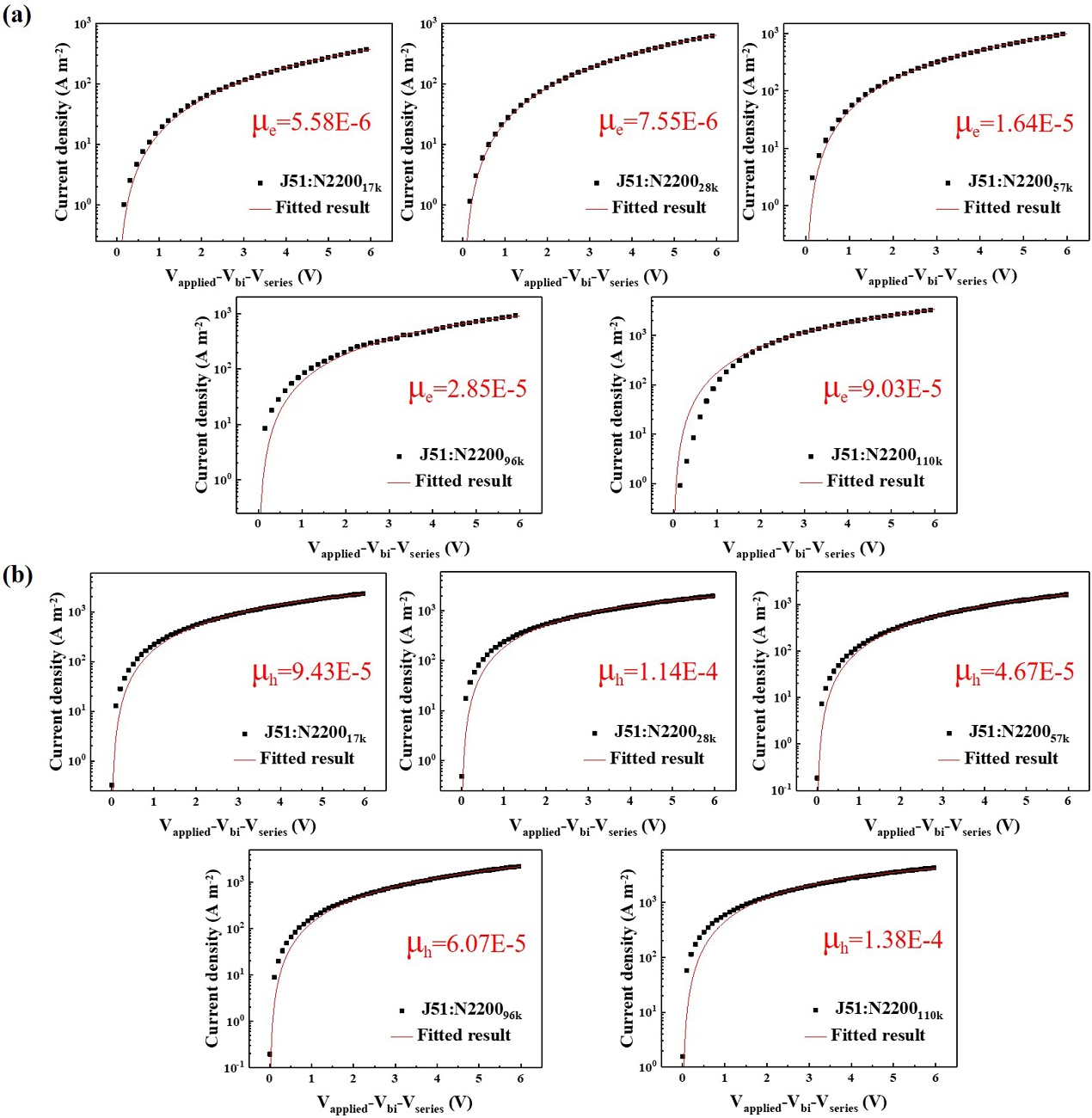


**Figure 2.** (a)The electron-only mobility (b) hole-only mobility of blend system with different MWs of N2200.


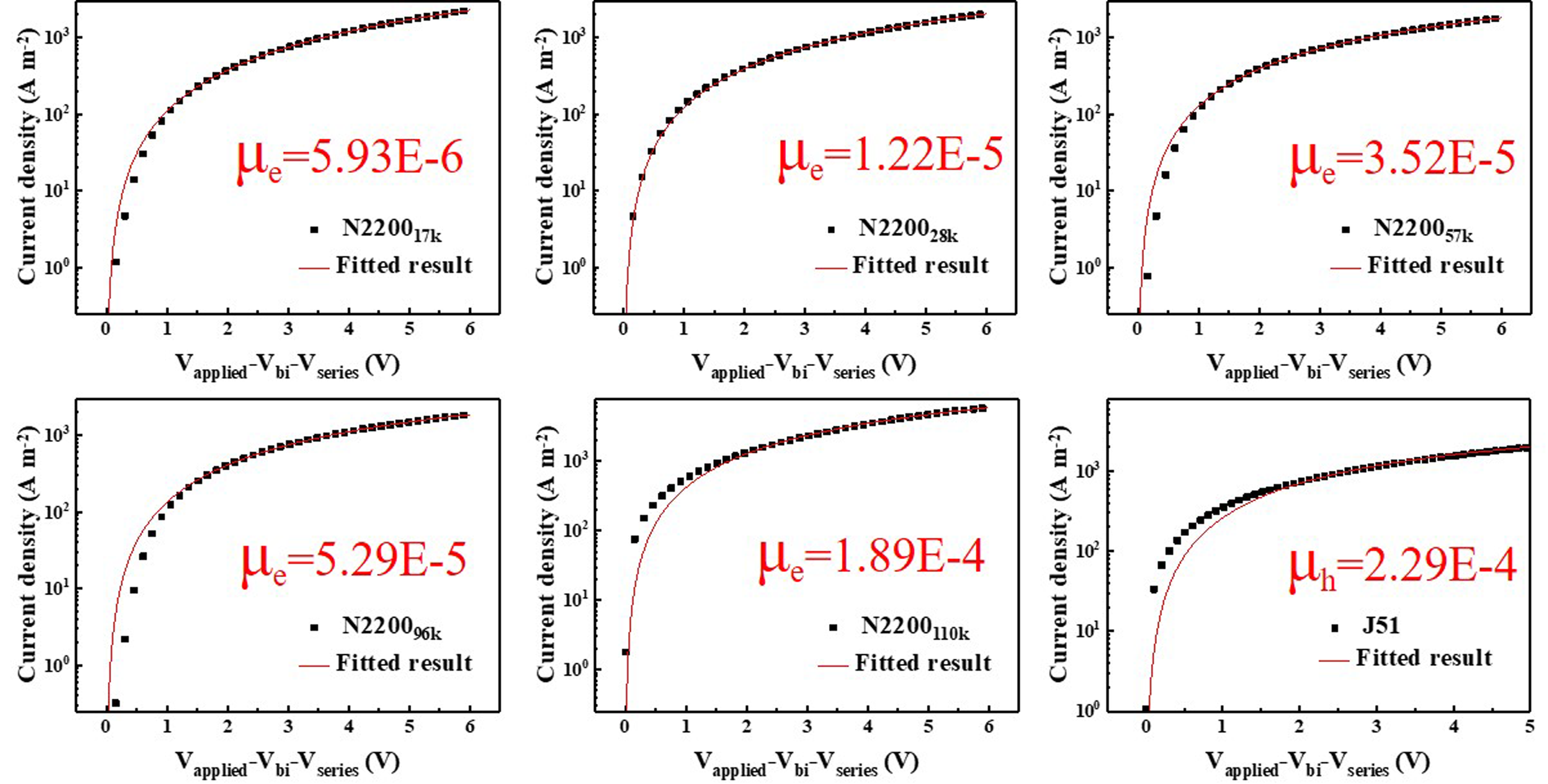


**Figure 3.** The electron-only and hole-only mobility for pure N2200 and J51.


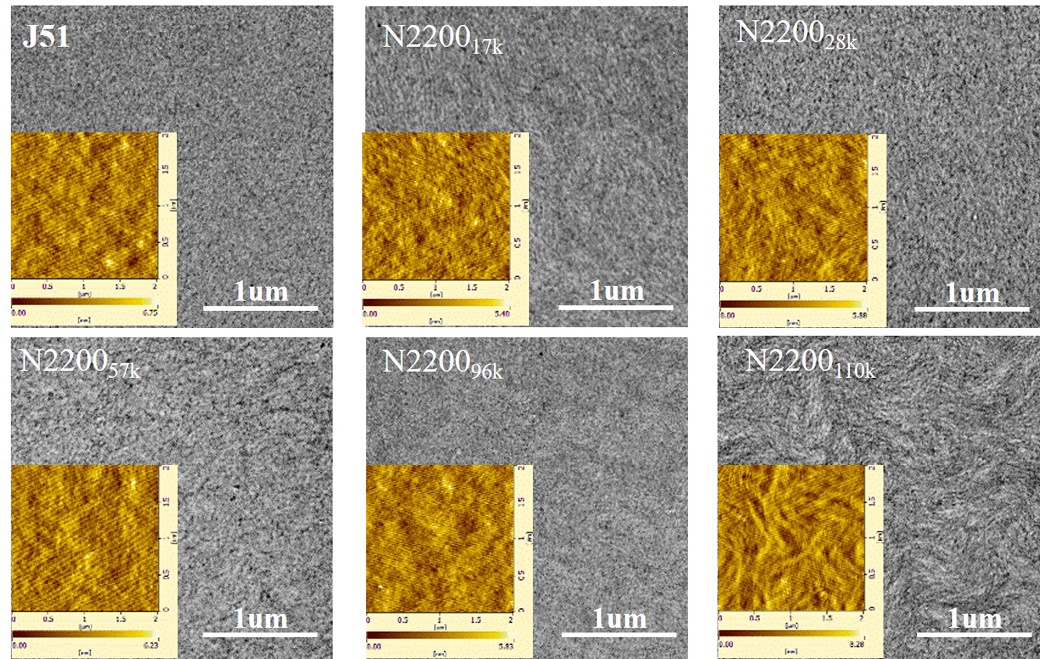


**Figure 4.** The morphology of pure component J51 and N2200 with different MWs.


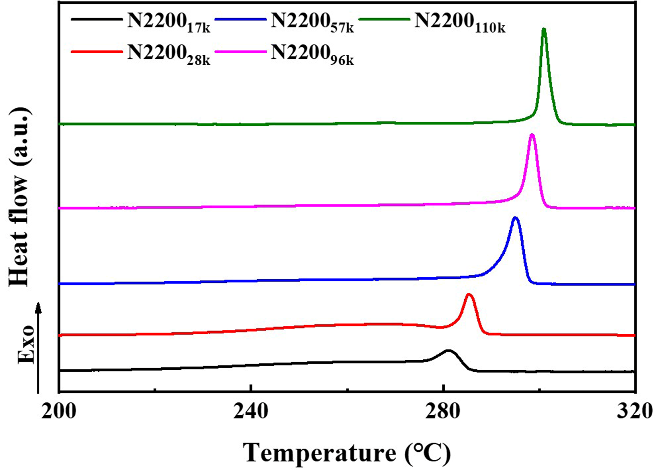


**Figure 5.** The first cooling cycles of DSC curves, the melting temperature (*T*_m_) as a function of N2200 MWs.

**Table 2.** The corresponding viscosity values with different N2200 MWs.

|  | **M_v_**  **(kDa)** | **η_N2200_**  **(mPa·S)** | **η_CF_**  **(mPa·S)** | **η_r_** | **η_sp_** | **[η]_k’_**  **_(dL/g)_** | **[η]_r_**  **_(dL/g)_** |
| --- | --- | --- | --- | --- | --- | --- | --- |
| N2200_17k_ | 33 | 0.64 | 0.61 | 1.05 | 0.05 | 0.12 | 0.12 |
| N2200_28k_ | 51 | 0.70 |  | 1.15 | 0.15 | 0.35 | 0.36 |
| N2200_57K_ | 150 | 1.30 |  | 2.13 | 1.13 | 2.01 | 2.12 |
| N2200_96k_ | 177 | 1.51 |  | 2.48 | 1.48 | 2.44 | 2.62 |
| N2200_110k_ | 205 | 3.58 |  | 5.87 | 4.87 | 5.40 | 6.36 |


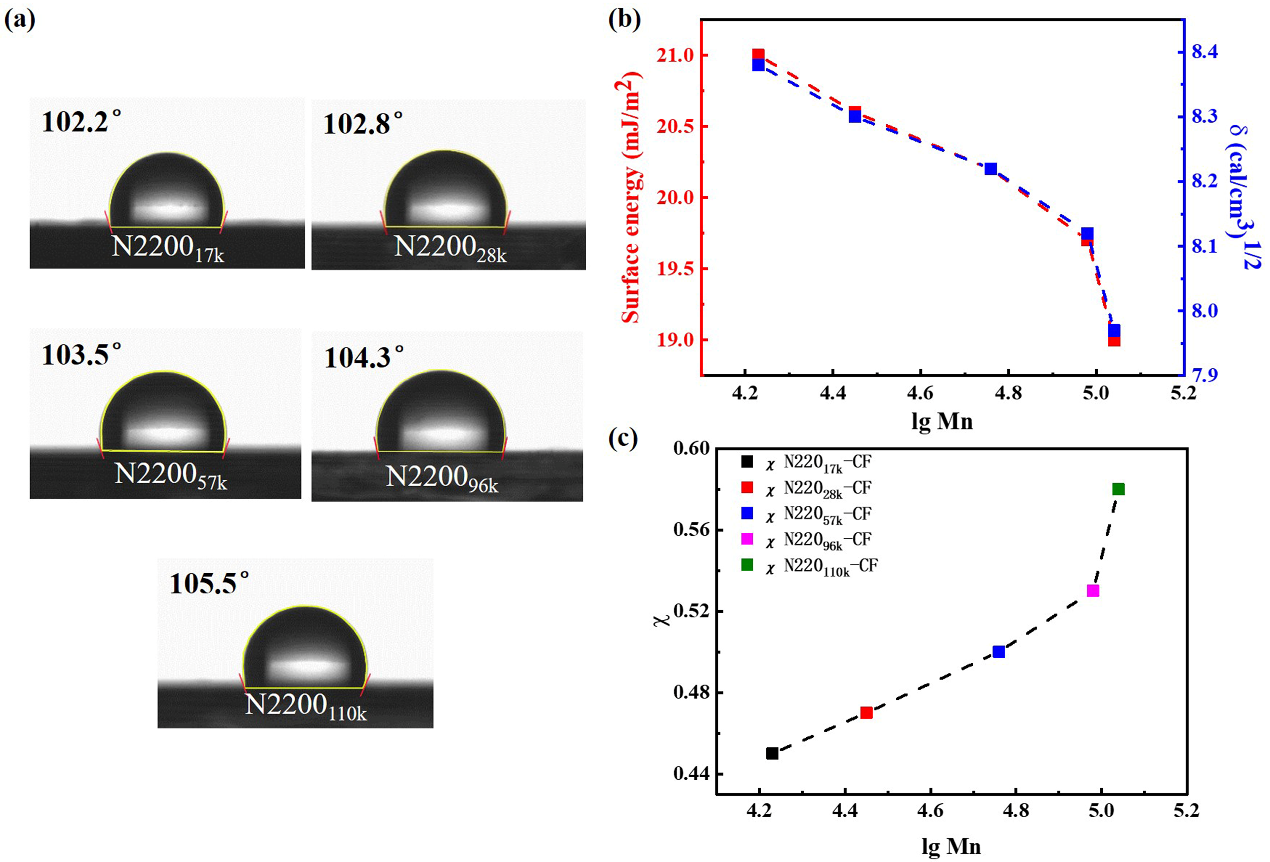


**Figure 6.** (a) Contact angle measurements (b) Surface energy and δ (c) Evaluated χ value for N2200 films at different MWs. (Calculate the χ between N2200 and chloroform using the contact angle experiment of water droplet method reported by Kim et al ^1^.)


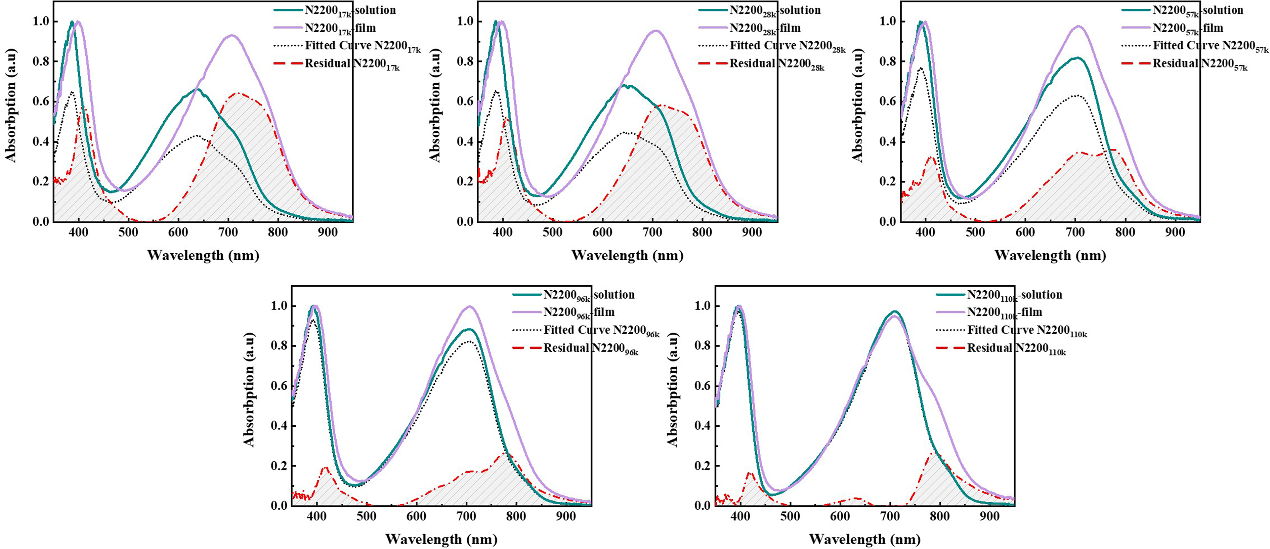


**Figure 7.** Using the Franck−Condon analysis of the absorption of different MWs of N2200.


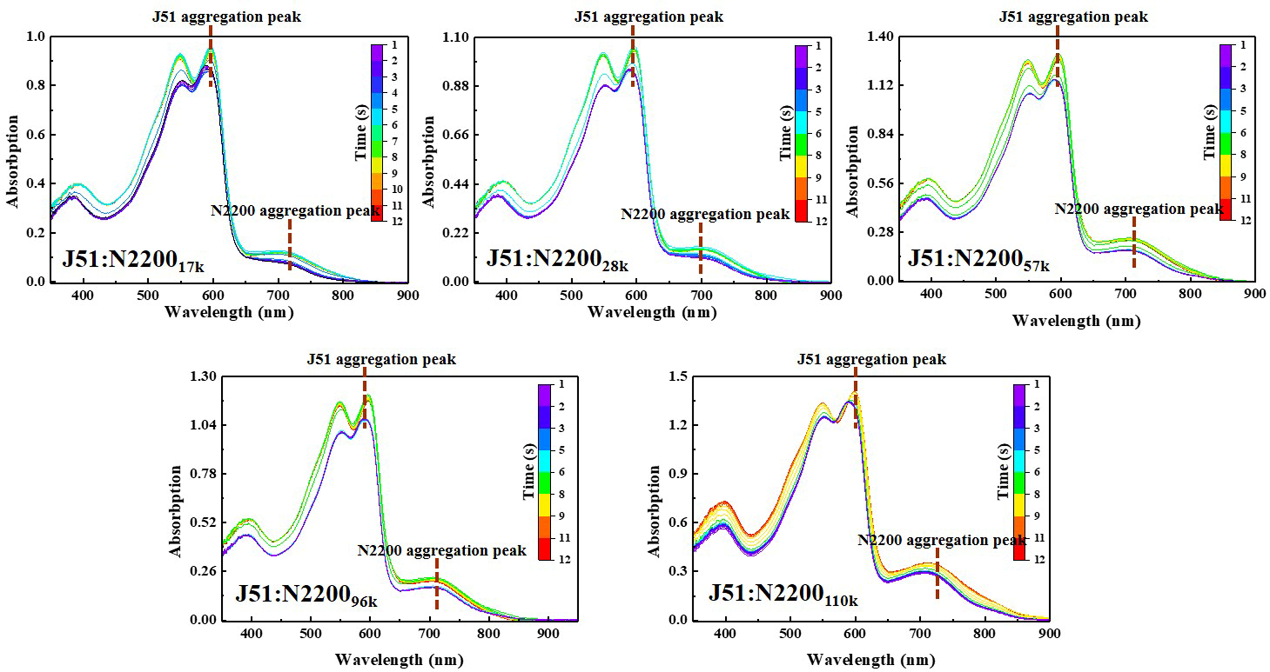


**Figure 8.** The in-situ film-forming absorption spectrum as a function of time with different N2200 MWs.

**References**

1. Kim, J. Y. (2019).Phase Diagrams of Binary Low Bandgap Conjugated Polymer Solutions and Blends. *Macromolecules.* doi:10.1021/acs.macromol.9b00477
